# Supplementary material for: Delayed room temperature phosphorescence enabled by phosphines
Source: Nat Commun. 2024 May 2;15:3705. doi: 10.1038/s41467-024-47888-z (PMC11066103; doi:10.1038/s41467-024-47888-z)
Supplement: Supplementary file 1 — Supplementary Information [file 41467_2024_47888_MOESM1_ESM.pdf]

**Supplementary Information**

# Delayed Room Temperature Phosphorescence Enabled by Phosphines

Guang Lu,[1] Jing Tan,[1] Hongxiang Wang,[1] Yi Man,[1] Shuo Chen,[1] Jing Zhang,[1] Chunbo Duan,[1] Chunmiao Han,[1] Hui Xu[1,*]

[1]Key Laboratory of Functional Inorganic Material Chemistry (Ministry of Education) & School of Chemistry and Material Science, Heilongjiang University, 74 Xuefu Road, Harbin 150080, P. R. China.

*Correspondence to: hxu@hlju.edu.cn (HX)

**Content**

Supplementary Note 1. Experimental Section ........ S2

Supplementary Note 2. Photophysical Properties ........ S5

Supplementary Note 3. Single-Crystal Structures ........ S13

Supplementary Note 4. Theoretical Simulation ........ S16

Supplementary Note 5. Time Encoded Security Design ........ S20

Supplementary Note 6. Structural Characteristics ........ S21

Supplementary Note 7. References ........ S28

# Supplementary Note 1. Experimental Section

*1. Materials and Instruments*

All the reagents and solvents were purchased from Aldrich and Acros companies and used without further purification. $^{1}$H NMR spectra were recorded using a Varian Mercury plus 400NB spectrometer relative to tetramethylsilane (TMS) as internal standard. Molecular masses were determined by a FINNIGAN LCQ Electro-Spraying Ionization-Mass Spectrometry (ESI-MS), or a MALDI-TOF-MS. The crystals suitable for single-crystal XRD analysis were obtained through vapor-phase diffusing *n*-hexane to dichloromethane solution (5 ml) of the materials (10 mg). All diffraction data were collected at 295 K on a Rigaku Xcalibur E diffractometer with graphite monochromatized Mo Kα (λ = 0.71073 Å) radiation in ω scan mode. All structures were solved by direct method and difference Fourier syntheses. Non-hydrogen atoms were refined by full-matrix least-squares techniques on F2 with anisotropic thermal parameters. The hydrogen atoms attached to carbons were placed in calculated positions with C−H = 0.93 Å and U(H) = 1.2Ueq(C) in the riding model approximation. All calculations were carried out with the SHELXL97 program. Absorption and photoluminescence (PL) emission spectra of the target compound were measured using a SHIMADZU UV-3150 spectrophotometer and a SHIMADZU RF-5301PC spectrophotometer, respectively. Phosphorescence spectra were measured in dichloromethane ($CH_2Cl_2$) using an Edinburgh FPLS 1000 fluorescence spectrophotometer at 77 K cooling by liquid nitrogen. The time decay spectra was measured using Time-Correlated Single Photon Counting (TCSPC) method with a picosecond hydrogen lamp for 100 ps-10 μs and a microsecond pulsed Xenon light source for 1 μs-10 s lifetime measurement, the synchronization photomultiplier for signal collection and the Multi-Channel Scaling Mode of the PCS900 fast counter PC plug-in card for data processing. Lifetime values were simulated by single exponential fitting function in Fluoracle software. For anti-counterfeiting and encryption applications, a circular ultraviolet flashlight (GET-104) was used as the excitation

source with a power of 3 W and the peak wavelength at 365 nm, whose spot diameter is about 2 cm at the distance of 10 cm above the samples.

*2. Pattern Preparation for Applications*

For multilevel information displays, materials featuring strong fluorescence and instant and delayed RTP with different lifetimes, namely DMAC-DPS, DCzSBr, DCzDBr and DCzSBrSP, were chosen for information encryption and anti-counterfeiting applications. DMAC-DPS is bis[4-(9,9-dimethyl-9,10-dihydroacridine)phenyl]sulfone with thermally activated delayed fluorescence at microsecond level chosen as emission background. Compared to another delayed RTP material DCzSBrSPO, RTP duration of DCzSBrSP is longer, which provides more time for distinguishing complicated information. More importantly, RTP intensity of DCzSBrSP is comparable to those of DCzSBr and DCzDBr, making information encryption mainly dependent on emission duration.

*3. Synthesis*

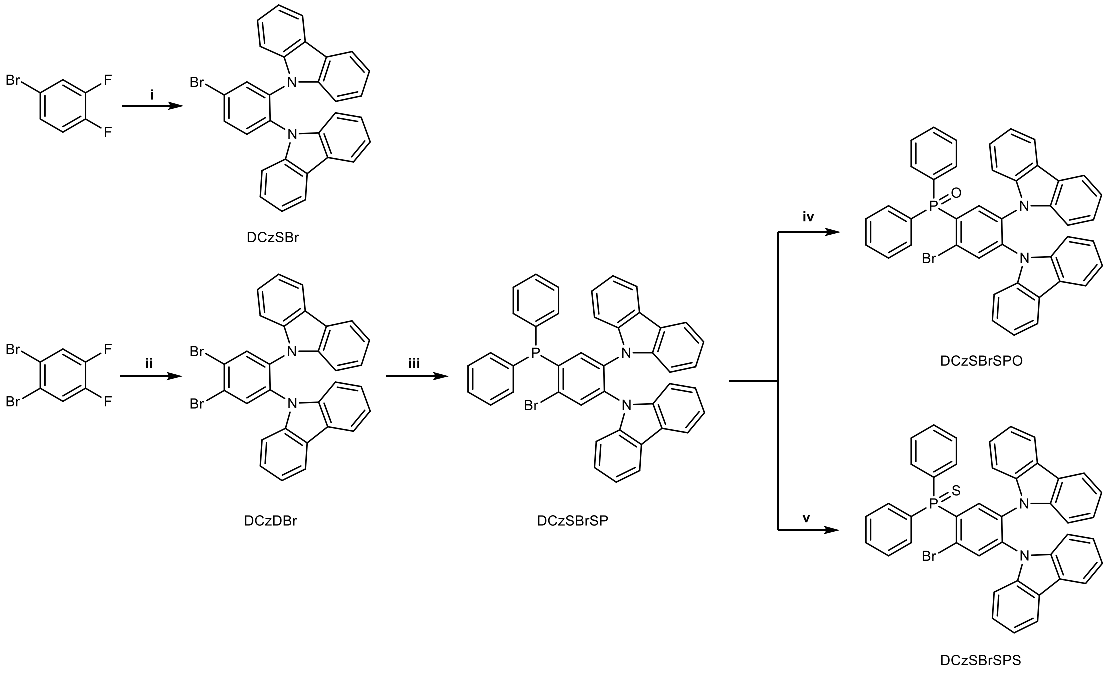


**Supplementary Figure 1**. Synthetic procedure of DCzSBr, DCzDBr, DCzSBrSP, DCzSBrSPO and DCzSBrSPS. i. Carbazole, KOH, DMSO, 140 ℃, 1.5 h; ii.

carbazole, $K_2CO_3$, DMSO, 150 °C, 12 h; iii. n-BuLi, $Ph_2PCl$, $Et_2O$ and THF, -120 °C, 12 h; iv. $H_2O_2$, DCM, 0°C, 4 h; v. $S_8$, $CHCl_3$, 60°C, 10h.

*Synthesis of (2-Bromo-4,5-di(9H-carbazol-9-yl)phenyl)diphenylphosphine sulfide (DCzSBrSPS)*

A mixture of DCzSBrSP (1.34g, 2 mmol) and $S_8$ (2.56 g, 1 mmol) in chloroform (10 mL) was stirred at 60°C for 10 h. After removing chloroform, the residue was purified with column chromatography to give white solid with a yield of 80% (1.13 g). $^1H$ NMR (TMS, $CDCl_3$, 400 MHz): $\delta$ = 8.188 (d, $J$ =3.6 Hz, 1H), 7.990-8.030 (m, 4H), 7.774 (d, $J$ =6.8 Hz, 2H), 7.719 (d, $J$ =6.4 Hz, 2H), 7.607 (d, $J$ =13.6 Hz, 1H), 7.485-7.572 (m, 6H), 6.937-7.133 ppm (m, 12H); $^{13}C$ NMR (TMS, $CDCl_3$, 101 MHz): $\delta$ =138.879, 138.767, 137.273, 137.245, 136.759, 136.677, 136.626, 136.505, 134.283, 133.429, 132.692, 132.560, 132.359, 132.252, 132.208, 132.180, 131.449, 130.575, 129.069, 128.941, 125.831, 125.598, 125.128, 125.068, 123.919, 123.661, 120.841, 120.572, 120.142, 120.018, 109.517, 109.205 ppm; $^{31}P$ NMR (TMS, $CDCl_3$, 162 MHz): $\delta$ = 45.798 ppm.

*4. Gaussian simulation*

Theoretical computations were carried out on the basis of the restricted and unrestricted formalism of Beck's three-parameter hybrid exchange functional[1] and Lee, and Yang and Parr correlation functional[2] (B3LYP). The optimization was also performed at the level of 6-31G(d,p), respectively, on the basis of single-crystal data. The fully optimized stationary points were further characterized by harmonic vibrational frequency analysis to ensure that real local minima had been found without imaginary vibrational frequency. The total energies were also corrected by zero-point energy both for the ground state and triplet state. Natural transition orbital (NTO) analysis was performed on the basis of optimized ground-state geometries at the same level.[3] The contours were visualized with Gaussview 5.0. All computations were performed using the Gaussian 09 package.[4]

## Supplementary Note 2. Photophysical Properties

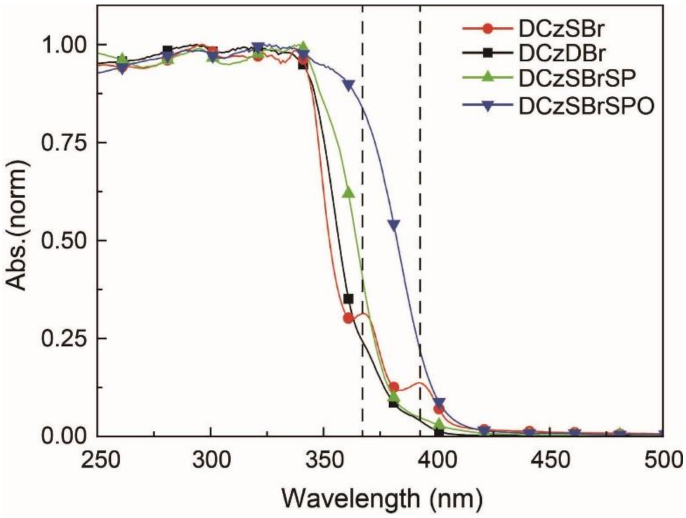


**Supplementary Figure 2**. Electronic absorption (abs.) spectra of DCzSBr, DCzDBr, DCzSBrSP and DCzSBrSPO powders. The dotted lines highlight the absorption bands of DCzSBr and DCzDBr in the range of 350-400 nm.

DCzSBr and DCzDBr reveal two absorption peaks in the range of 350-400 nm, which can be attributed to $S_0 \rightarrow T_1$ transitions of stabilized carbazole groups in DCzSBr and DCzDBr powders, according to Franck-Condon Principle (Figure S1). Therefore, it is rational that phosphorescence emissions of DCzSBr and DCzDBr have strong correlations with these excitation bands (Figure 2b). In contrast, absorption bands of aryl phosphine groups in DCzSBrSP and DCzSBrSPO also locate in this region, which overlap with the triplet absorptions of their carbazole groups. As consequence, their emission-excitation mapping shows broad and structure-less contours in this region.

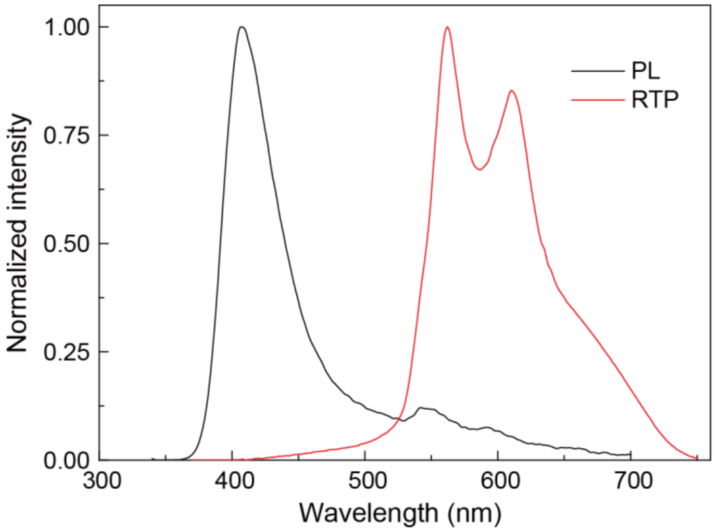


**Supplementary Figure 3**. Steady-state photoluminescence (PL, black line) and time-resolved RTP (red line) spectra of DCzSBrSPS powder under ambient condition and excitation at 330 nm.

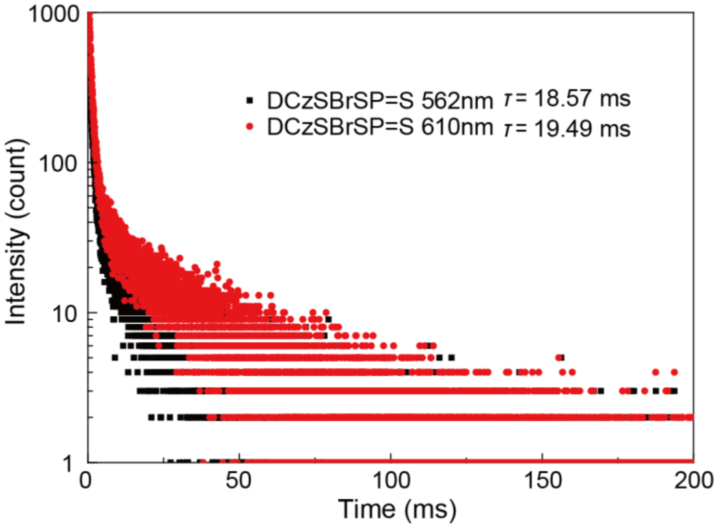


**Supplementary Figure 4**. Time decays of DCzSBrSPS powder at 562nm and 610nm, respectively, under excitation at 360nm.

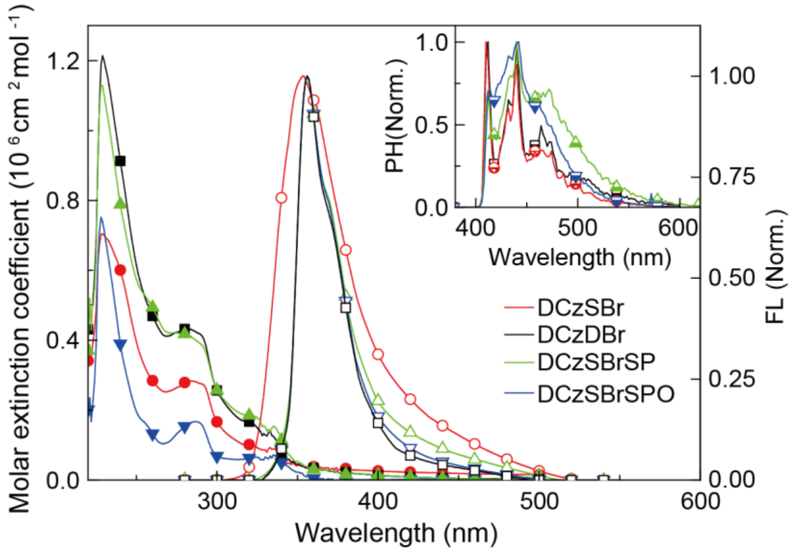


**Supplementary Figure 5**. Electronic absorption and steady-state PL spectra of DCzSBr, DCzDBr, DCzSBrSP and DCzSBrSPO in dilute $CH_2Cl_2$ ($1.0\times10^{-6}$ M). The inset is single-molecular phosphorescence spectra recorded at 77K after a delay of 10 microseconds.

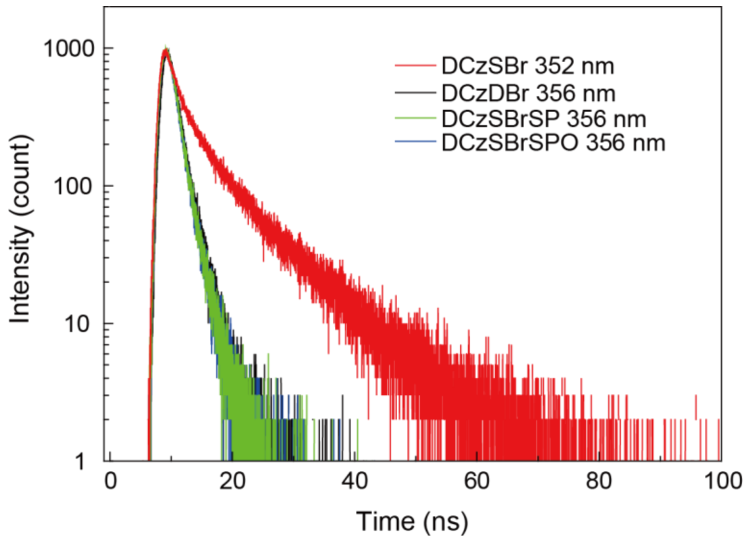


**Supplementary Figure 6**. Lifetime decays of DCzSBr, DCzDBr, DCzSBrSP and DCzSBrSPO in dilute $CH_2Cl_2$ ($1.0\times10^{-5}$ M) under ambient condition.

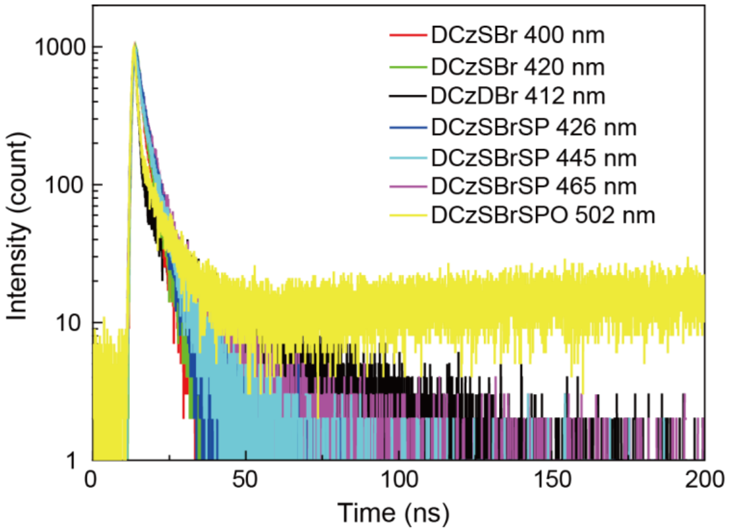


**Supplementary Figure 7**. Lifetime decays of fluorescent components from DCzSBr, DCzDBr, DCzSBrSP and DCzSBrSPO powders at nanosecond range. The tail of DCzSBrSPO at 502 nm can be attributed to its single-molecular phosphorescence at microsecond scale as shown in Supplementary Figure 11.

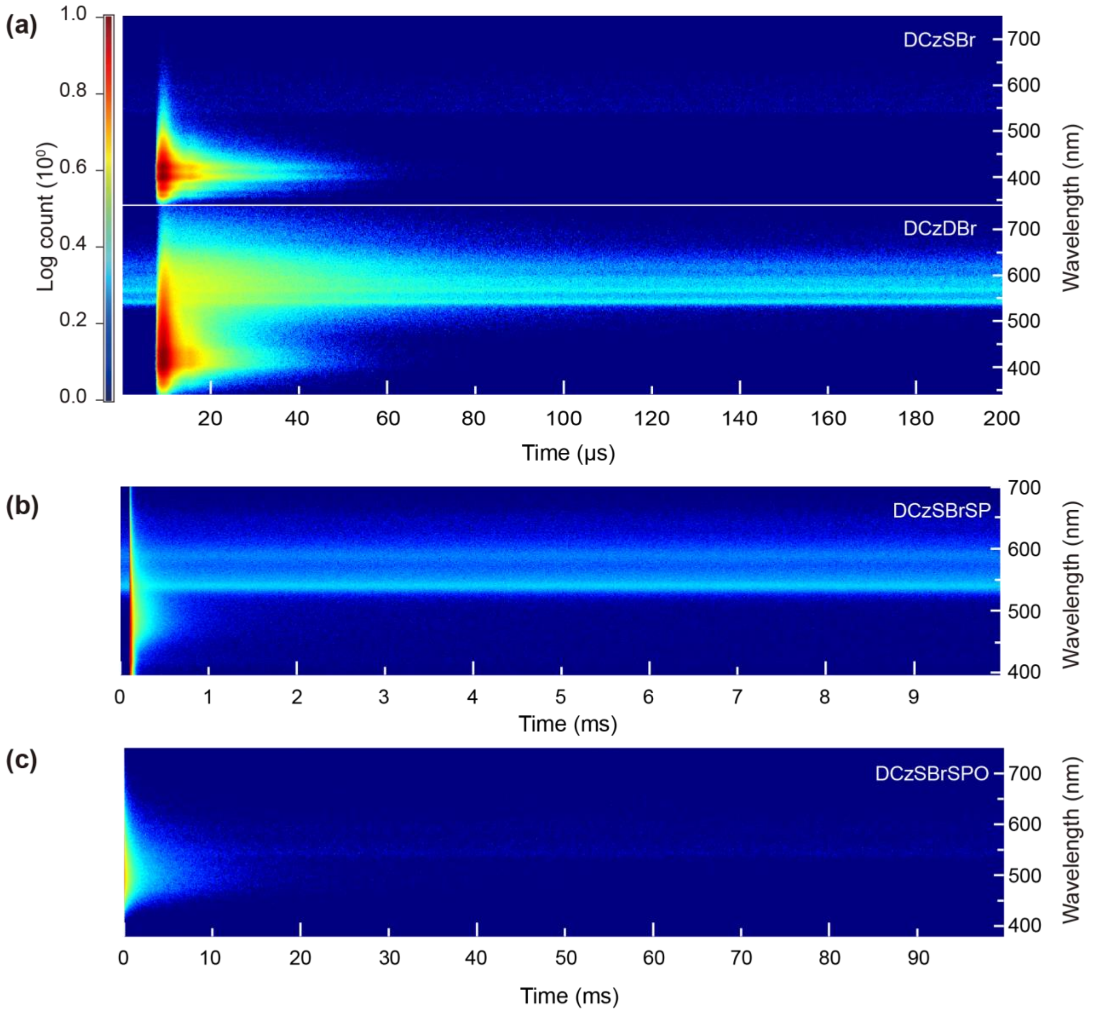


**Supplementary Figure 8**. Time-resolved emission spectra (TRES) of (a) DCzSBr and DCzDBr, (b) DCzSBrSP and (c) DCzSBrSPO powders. The excitation wavelength was 350nm for DCzSBr and 390nm for DCzDBr, DCzSBrSP and DCzSBrSPO.

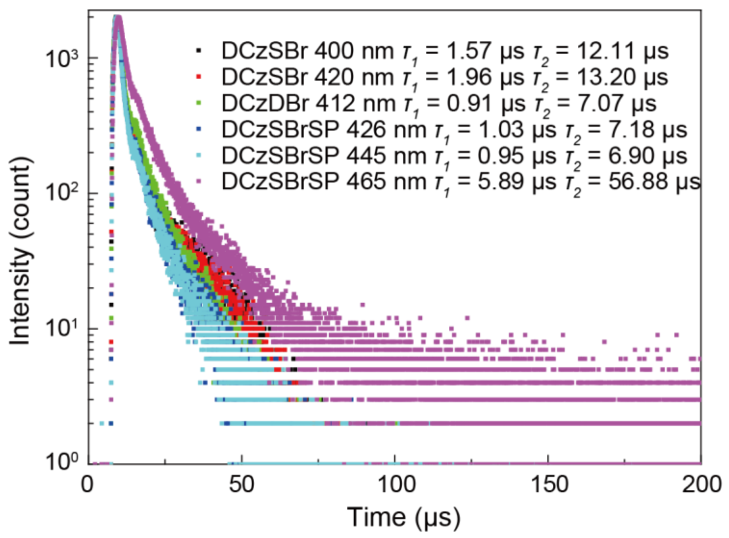


**Supplementary Figure 9**. Lifetime decays of single-molecular phosphorescence from DCzSBr, DCzDBr and DCzSBrSP powders at microsecond range.

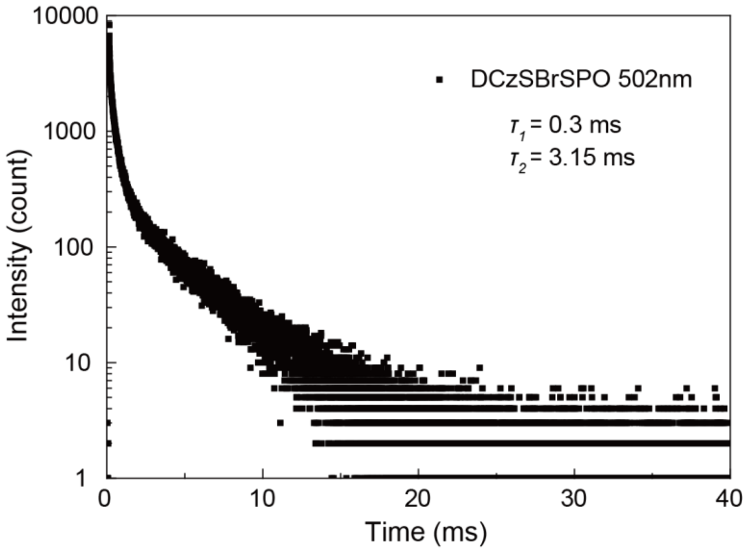


**Supplementary Figure 10**. Lifetime decay of single-molecular phosphorescence from DCzSBrSPO powder at millisecond range, which consists of two phosphorescence components with lifetimes of 0.3 and 3.15 ms, respectively.

**Supplementary Table 1**. Photophysical properties of DCzSBr, DCzDBr, DCzSBrSP and DCzSBrSPO powders

| Compound | Emission (nm) | | Lifetime | | RTP QE (%)[f] |
|---|---|---|---|---|---|
| | FL[a] | RTP[c] | $\tau_{FL}$ (ns)[d] | $\tau_{RTP}$ (ms)[e] | |
| DCzSBr | 400[b]<br>420[b] | 540 [b]<br>590 [b] | 2.92<br>3.01 | 116.27<br>111.55 | 6.92 |
| DCzDBr | 412[b] | 540 [b]<br>590 [b] | 0.99/13.97 | 79.77<br>73.21 | 24.2 |
| DCzSBrSP | 465[b]<br>426[b]<br>445[b] | 540 [b]<br>590 [b] | 3.58<br>3.69<br>4.30 | 742.10<br>737.09 | 1.49 |
| DCzSBrSPO | 504[b] | 540 [b]<br>590 [b] | -- | 502.57<br>490.45 | 0.95 |

[a] Fluorescence; [b] excited at 390 nm; [c] room temperature phosphorescence (RTP); [d] fluorescence lifetime; [e] RTP lifetime; [g] RTP quantum efficiency (QE).

**Supplementary Table 2**. Physical properties of DCzSBr, DCzDBr, DCzSBrSP and DCzSBrSPO in dichloromethane solutions

| Compound | $\lambda_{Abs}$ [a] (nm) | $\lambda_{PL}$ [a,b] (nm) | $\tau$ [a,b,c] (ns) | $S_1$ [d] (eV) | $T_1$ [e] (eV) | $\Delta E_{ST}$ [f] (eV) | HOMO[g] (eV) | LUMO[g] (eV) |
|---|---|---|---|---|---|---|---|---|
| DCzSBr | 332, 322, 283, 228 | 352 | 2.14/9.18 | 3.54 | 3.02 | 0.52 | -5.398 | -0.910 |
| DCzDBr | 333, 320, 280, 229 | 356 | 1.76 | 3.51 | 3.01 | 0.50 | -5.511 | -1.155 |
| DCzSBrSP | 335, 322, 274, 228 | 356 | 1.62 | 3.48 | 3.01 | 0.47 | -5.322 | -1.133 |
| DCzSBrSPO | 337, 325, 286, 228 | 356 | 1.63 | 3.57 | 3.01 | 0.56 | -5.435 | -1.321 |

[a] In dichloromethane solution ($10^{-6}$ mol $L^{-1}$); [b] excited at 320 nm; [c] emission lifetime; [d] estimated according to the absorption edges; [e] calculated according to the 0-0 transitions of phosphorescence spectra; [f] singlet-triplet splitting energy; [g] DFT calculated results.

## Supplementary Note 3. Single-Crystal Structures

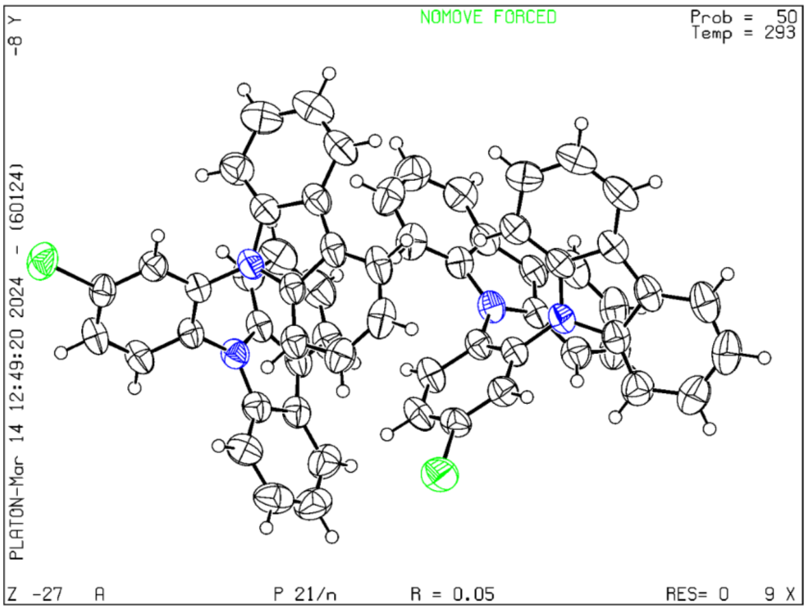


**Supplementary Figure 11**. ORTEP diagram of DCzSBr with 50% probability ellipsoids.

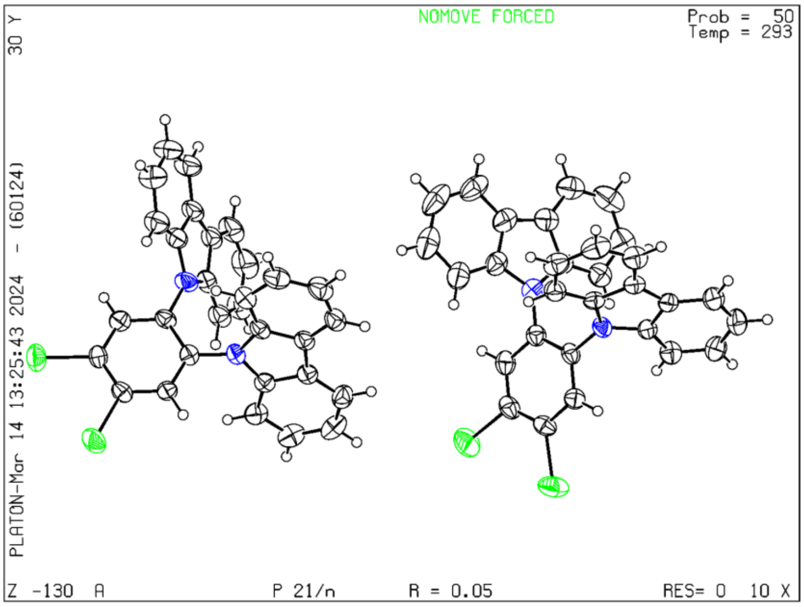


**Supplementary Figure 12**. ORTEP diagram of DCzDBr with 50% probability ellipsoids.

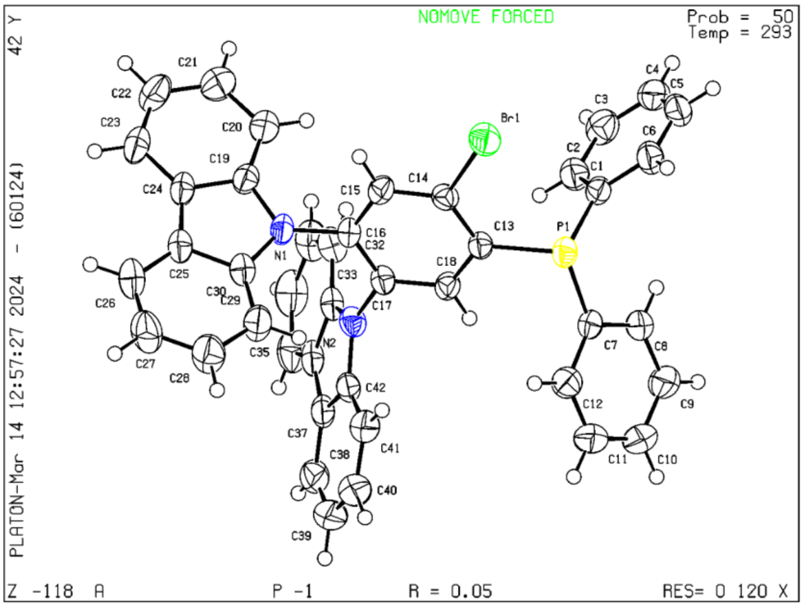


**Supplementary Figure 13**. ORTEP diagram of DCzSBrSP with 50% probability ellipsoids.

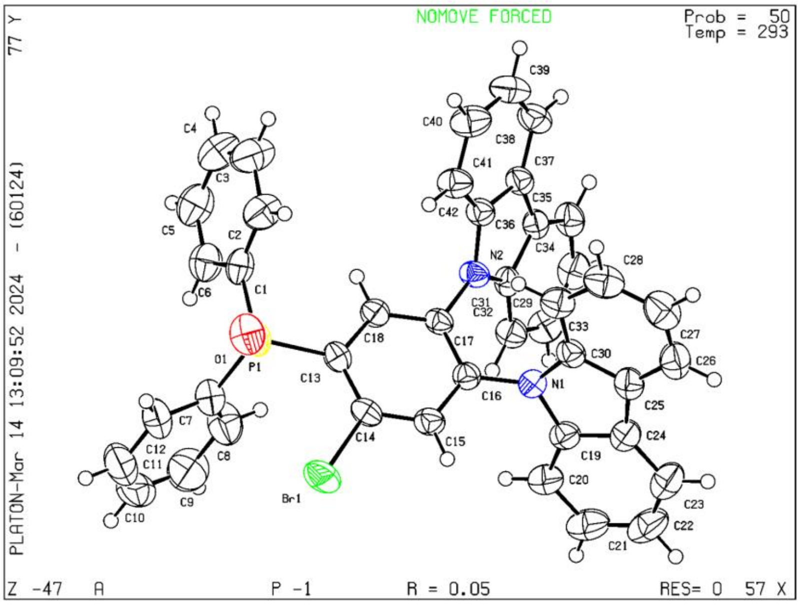


**Supplementary Figure 14**. ORTEP diagram of DCzSBrSPO with 50% probability ellipsoids.

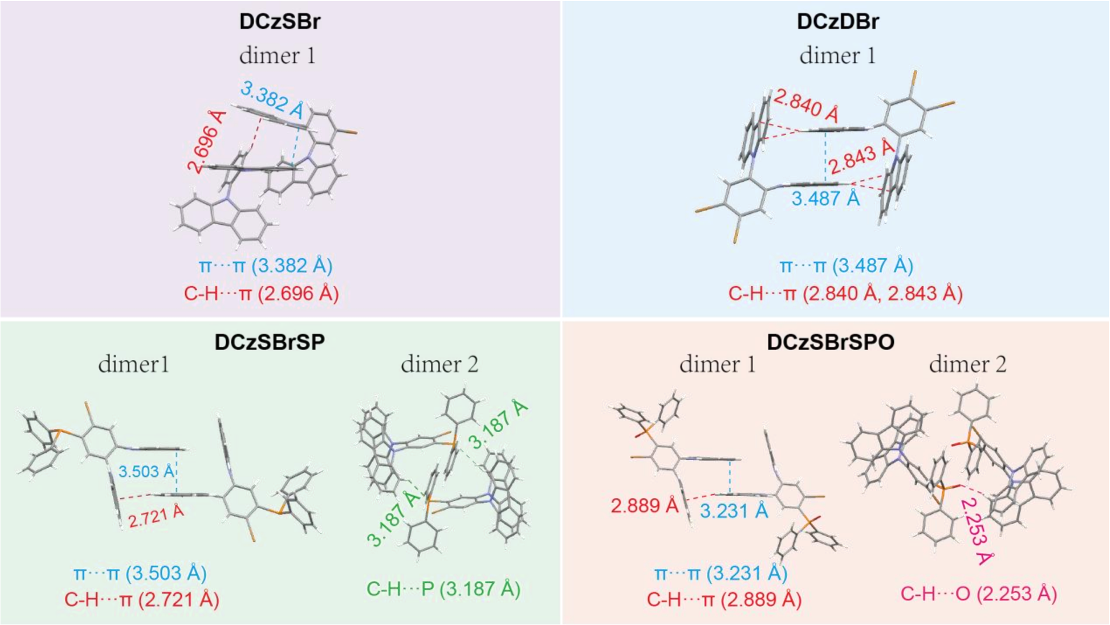


**Supplementary Figure 15**. Single-crystal packing diagrams of DCzSBr, DCzDBr, DCzSBrSP and DCzSBrSPO.

## Supplementary Note 4. Theoretical Simulation

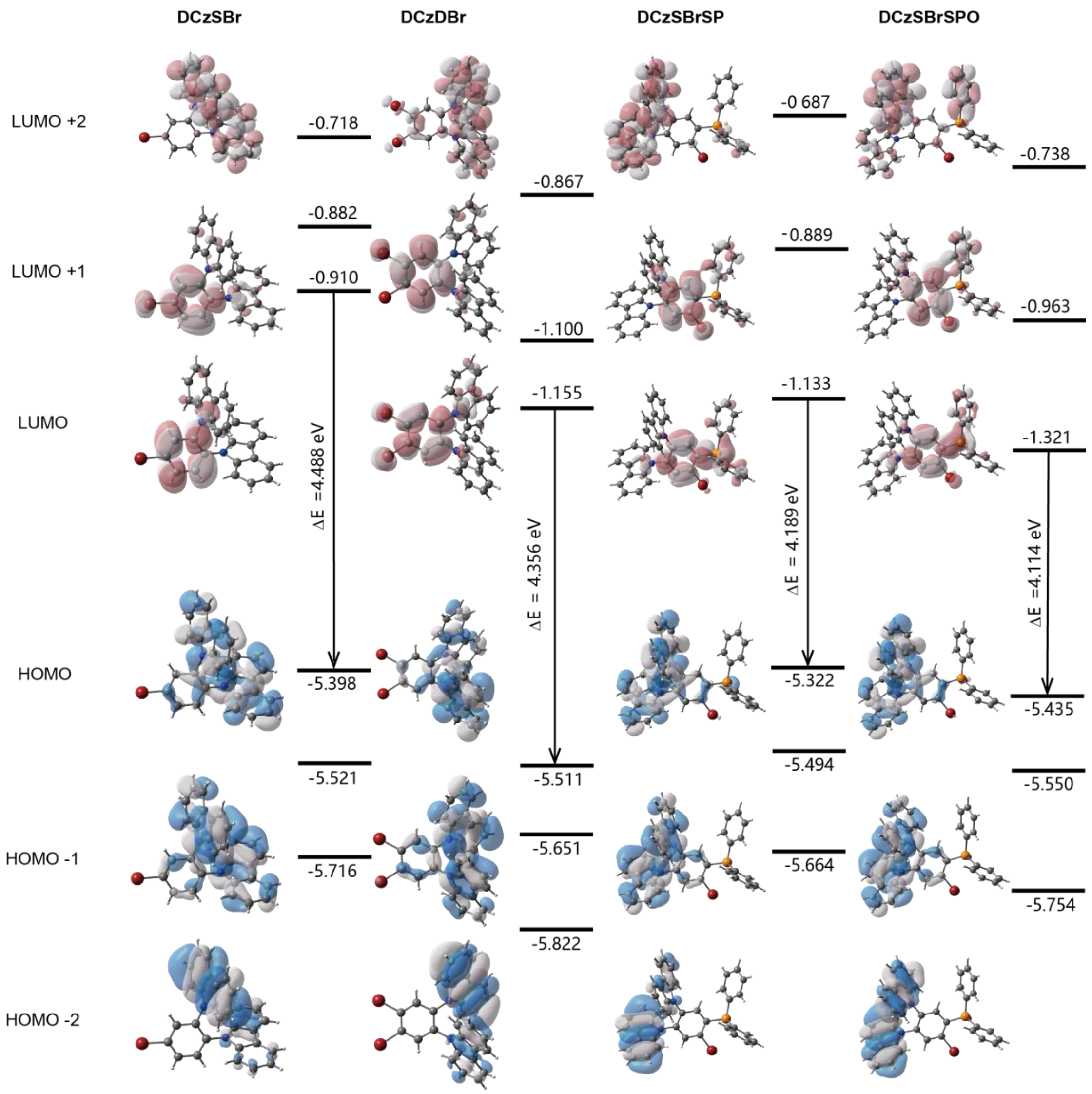


**Supplementary Figure 16**. Contours and energy levels of the frontier molecular orbitals for ground-state DCzSBr, DCzDBr, DCzSBrSP and DCzSBrSPO. The HOMO and the LUMO refer to the highest occupied and lowest unoccupied molecular orbitals, respectively.

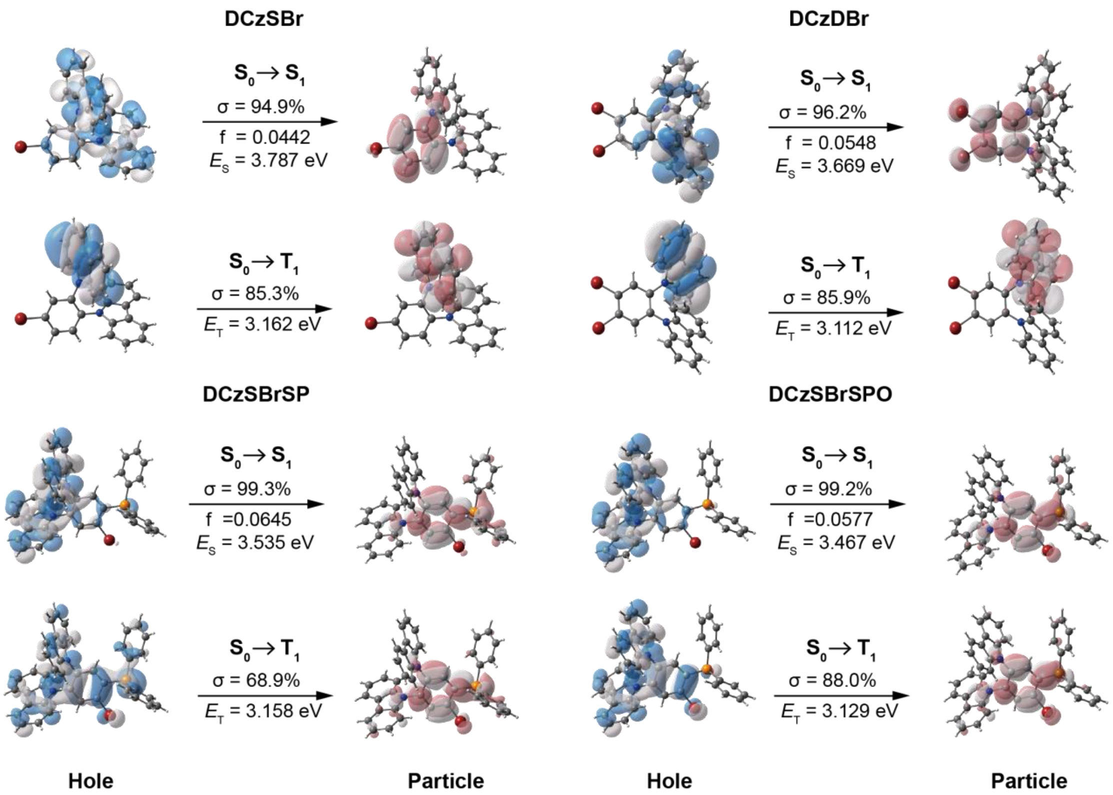


**Supplementary Figure 17**. Contours of "hole" and "particle" and transition parameters for singlet and triplet excitations of DCzSBr, DCzDBr, DCzSBrSP and DCzSBrSPO simulated with natural transition orbital (NTO) analysis. $S_0$, $S_1$ and $T_1$ refer to ground state, and the first singlet and the first triplet excited states. E, f, and σ refer to excited-state energy level, oscillator strength and contribution weight, respectively. The subscripts of "S" and "T" correspond to singlet and triplet states.

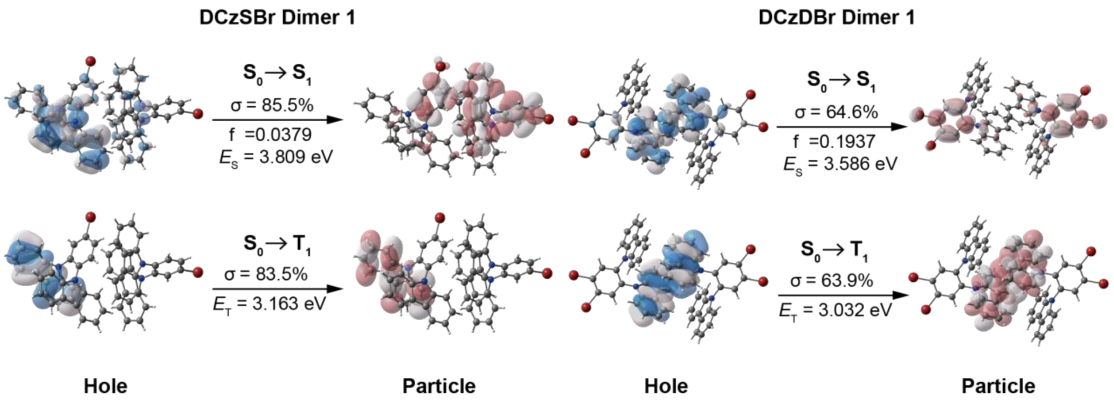


**Supplementary Figure 18**. Contours of “hole” and “particle” and transition parameters for singlet and triplet excitations of dimers for DCzSBr and DCzDBr simulated with natural transition orbital (NTO) analysis. $S_0$, $S_1$ and $T_1$ refer to ground state, and the first singlet and the first triplet excited states. E, f, and σ refer to excited-state energy level, oscillator strength and contribution weight, respectively. The subscripts of “S” and “T” correspond to singlet and triplet states.

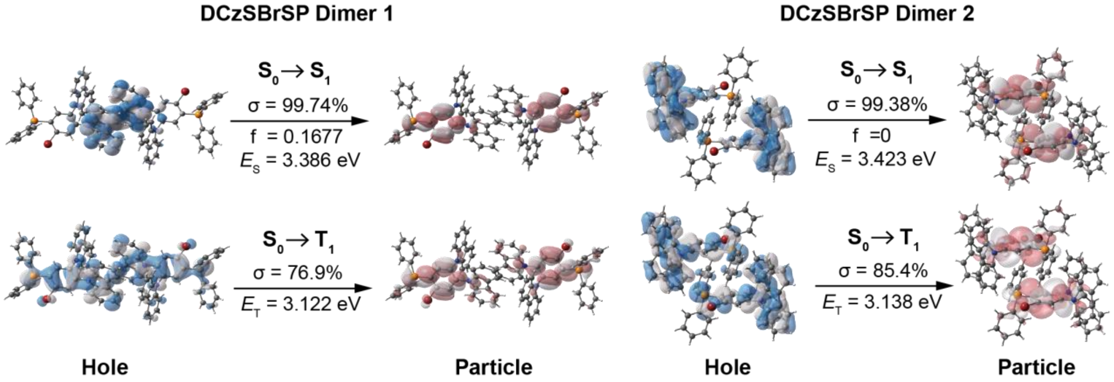


**Supplementary Figure 19**. Contours of “hole” and “particle” and transition parameters for singlet and triplet excitations of DCzSBrSP dimers 1 and 2 simulated with natural transition orbital (NTO) analysis. $S_0$, $S_1$ and $T_1$ refer to ground state, and the first singlet and the first triplet excited states. E, f, and σ refer to excited-state energy level, oscillator strength and contribution weight, respectively. The subscripts of “S” and “T” correspond to singlet and triplet states.

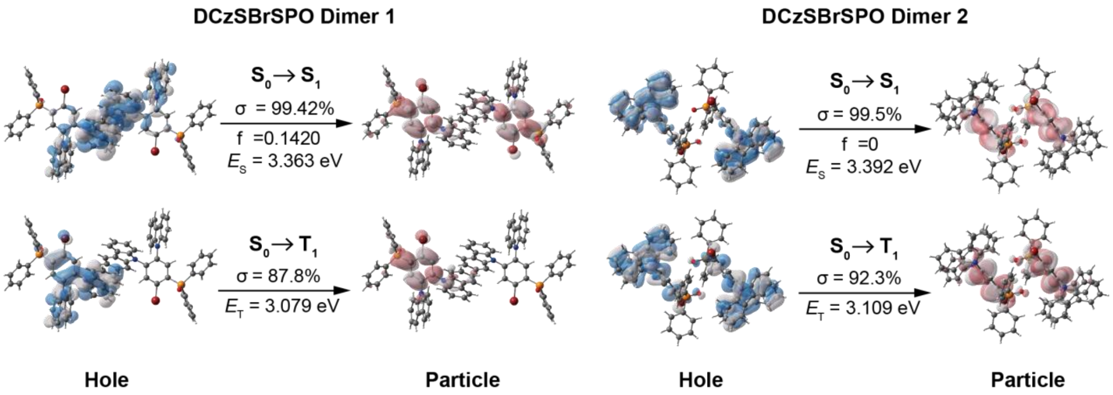


**Supplementary Figure 20**. Contours of "hole" and "particle" and transition parameters for singlet and triplet excitations of DCzSBrSPO dimers 1 and 2 simulated with natural transition orbital (NTO) analysis. $S_0$, $S_1$ and $T_1$ refer to ground state, and the first singlet and the first triplet excited states. E, f, and σ refer to excited-state energy level, oscillator strength and contribution weight, respectively. The subscripts of "S" and "T" correspond to singlet and triplet states.

## Supplementary Note 5. Time Encoded Security Design

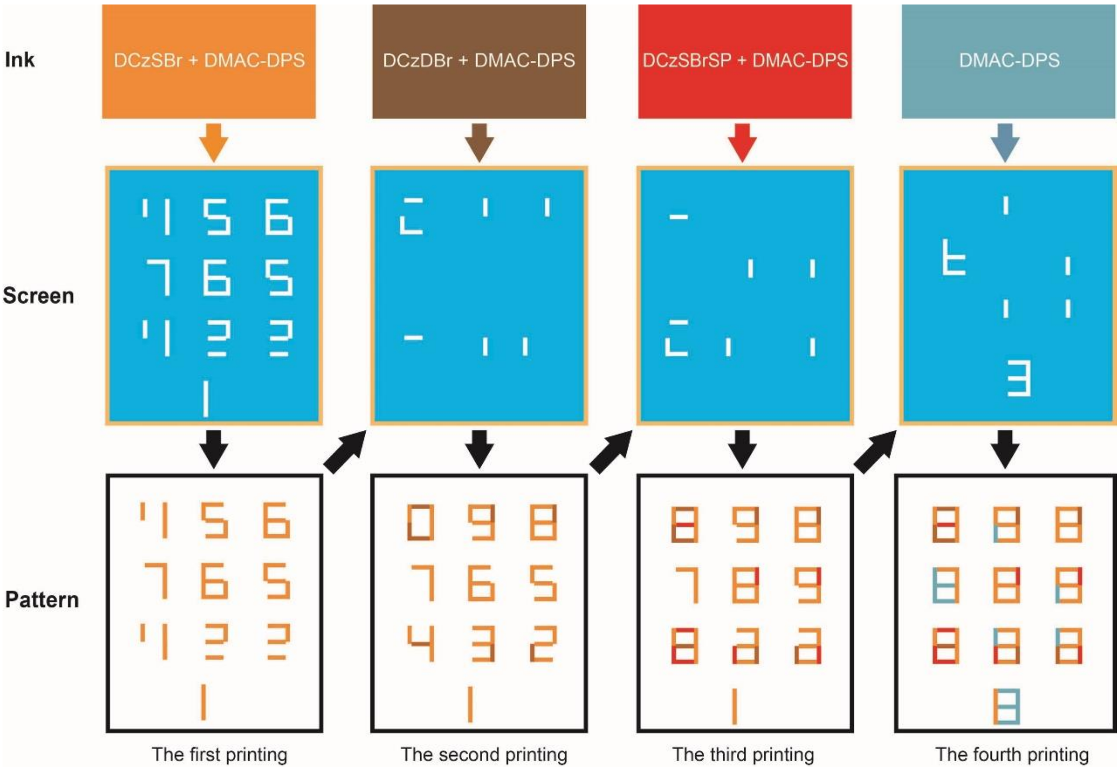


**Supplementary Figure 21.** Flow diagram of printing the password panel for non-linear time encoded security application. Black arrows indicate the procedure.

# Supplementary Note 6. Structural Characteristics

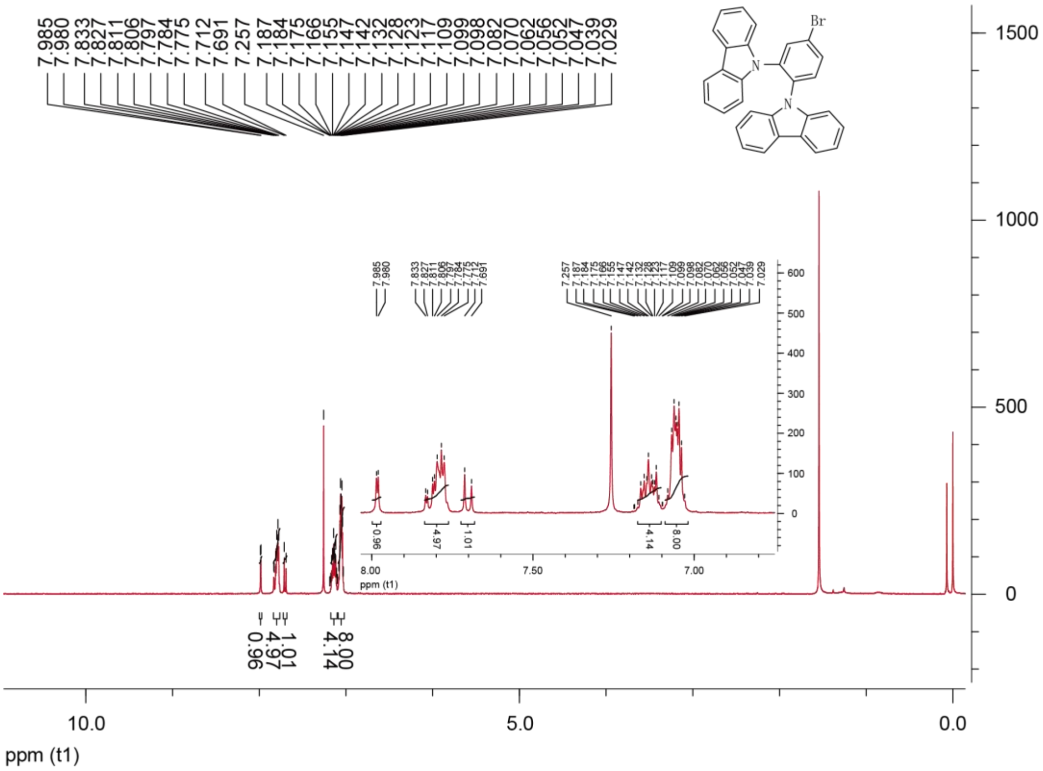


**Supplementary Figure 22**. $^{1}$H NMR (400 MHz) spectrum of DCzSBr in $CDCl_3$.

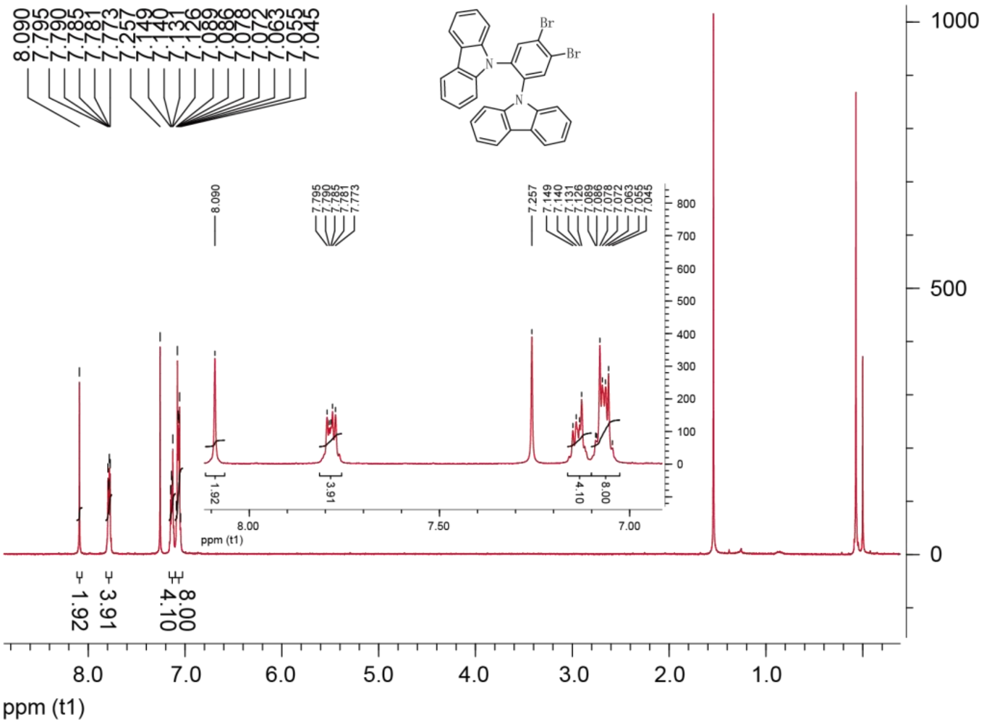


**Supplementary Figure 23**. $^{1}$H NMR (400 MHz) spectrum of DCzDBr in $CDCl_3$.

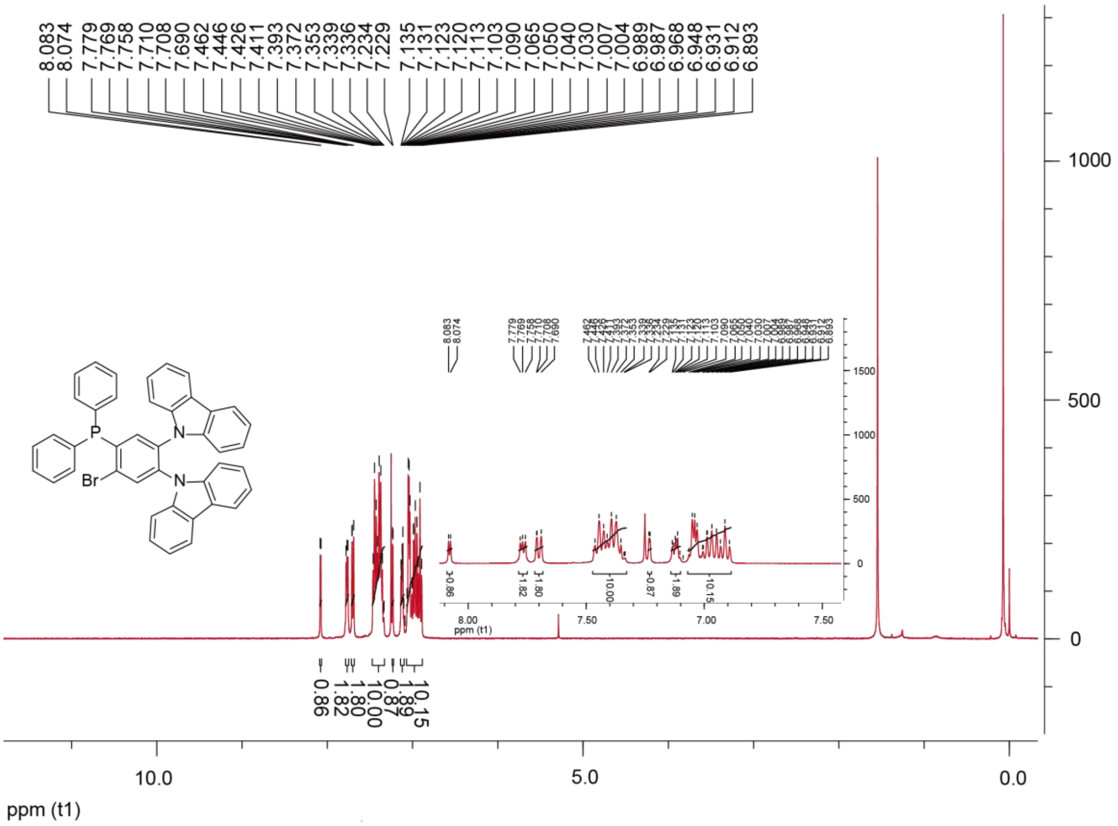


**Supplementary Figure 24**. $^{1}$H NMR (400 MHz) spectrum of DCzSBrSP in $CDCl_3$.

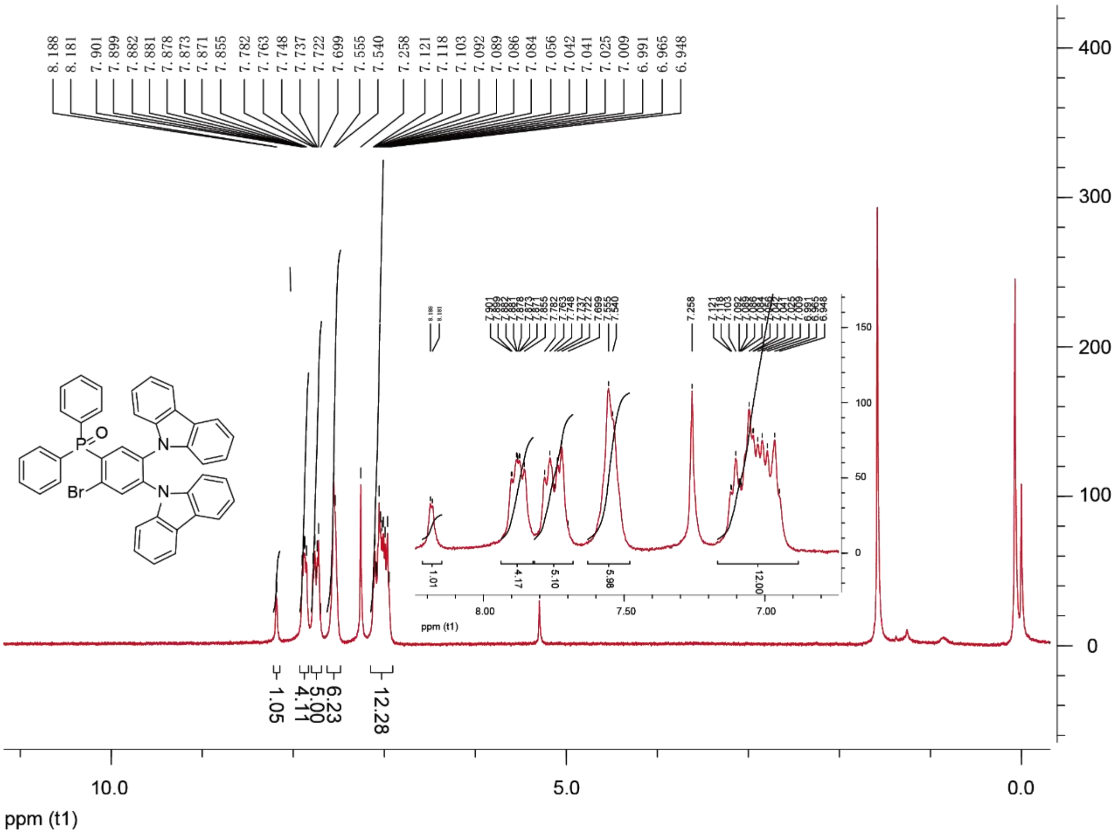


**Supplementary Figure 25**. $^{1}$H NMR (400 MHz) spectrum of DCzSBrSPO in $CDCl_3$.

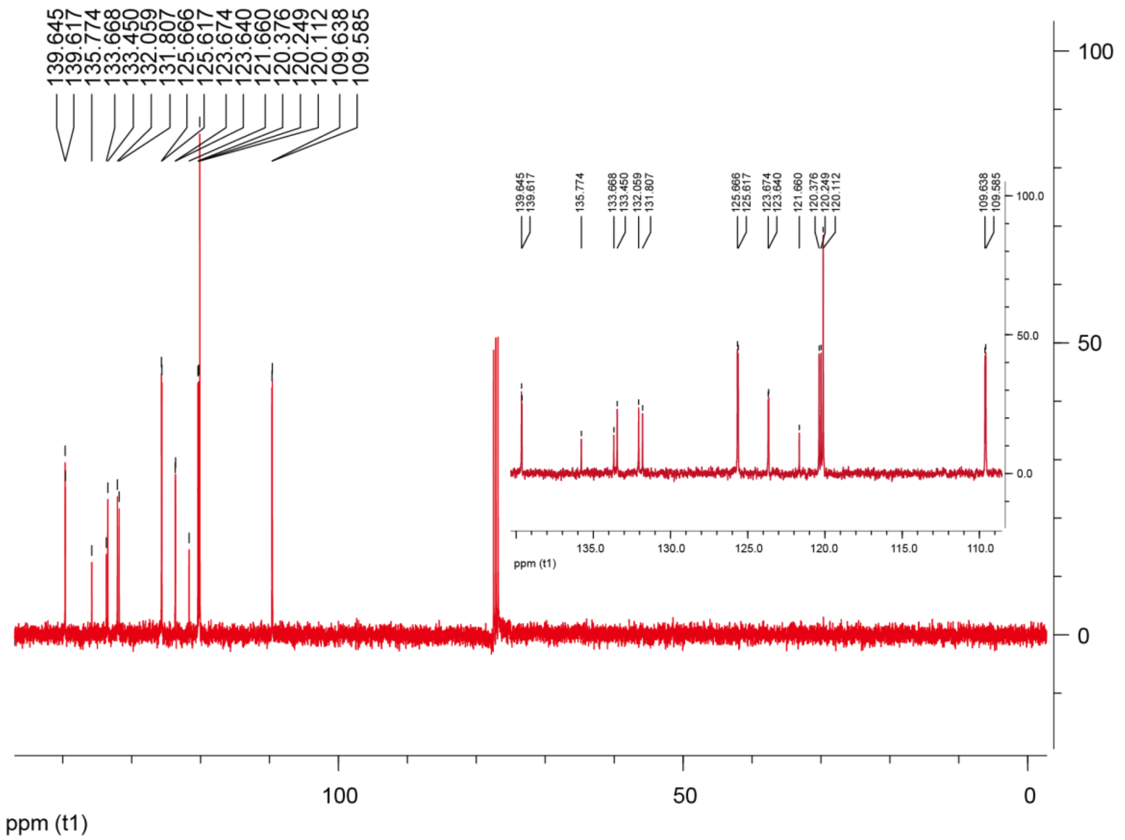


**Supplementary Figure 26**. $^{13}C$ NMR (101 MHz) spectrum of DCzSBr in $CDCl_3$.

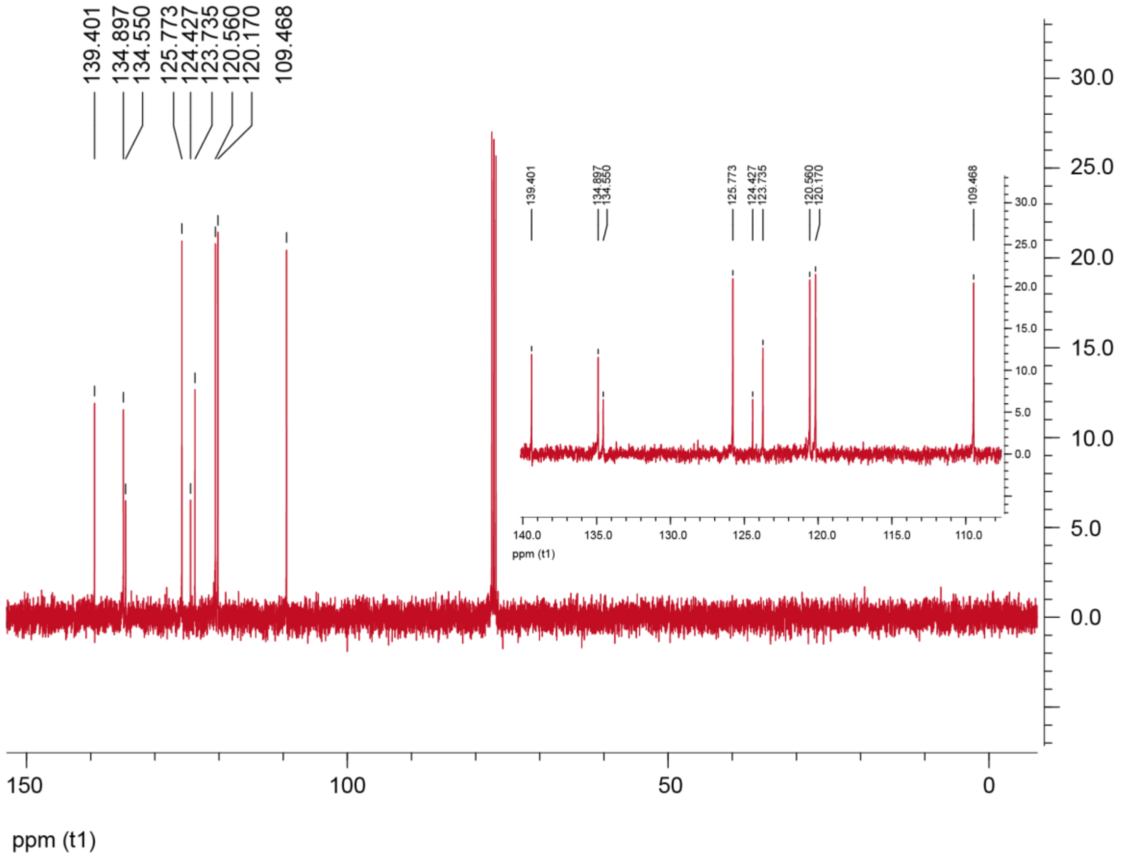


**Supplementary Figure 27**. $^{13}C$ NMR (101 MHz) spectrum of DCzDBr in $CDCl_3$.

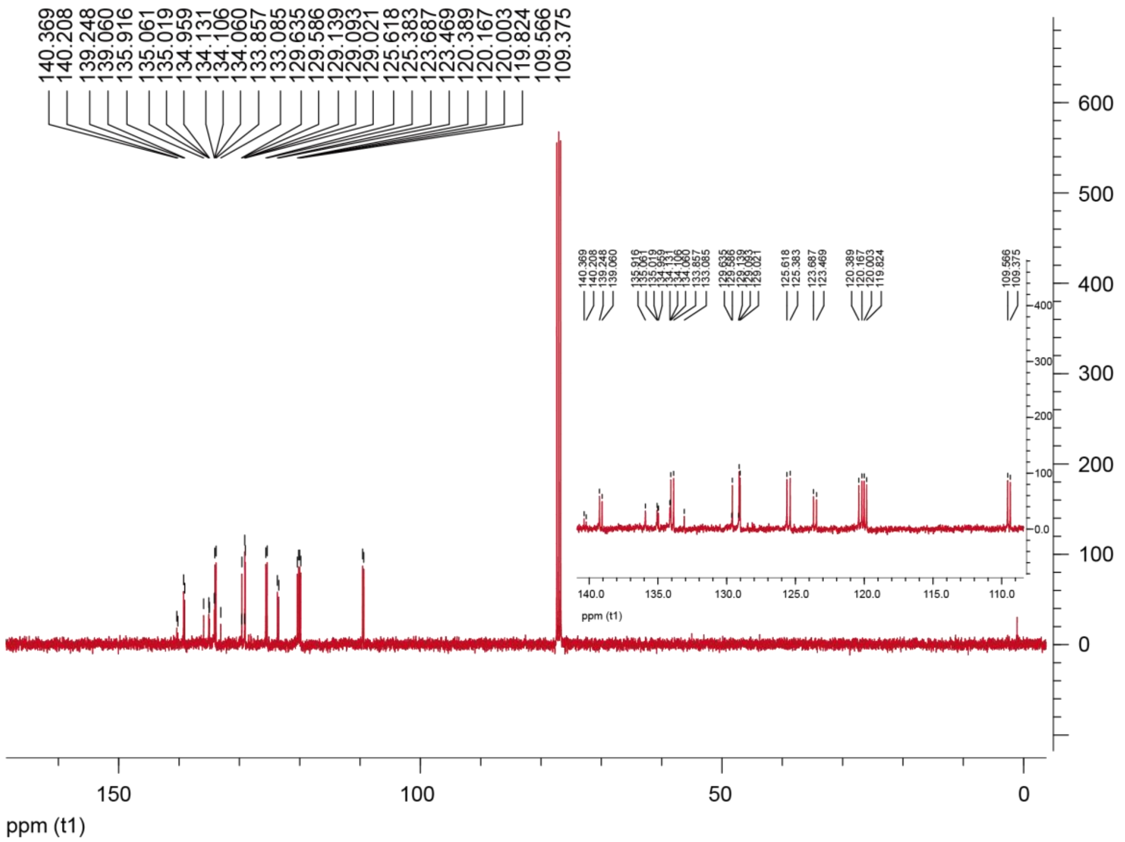


**Supplementary Figure 28**. $^{13}$C NMR (101 MHz) spectrum of DCzSBrSP in $CDCl_3$.

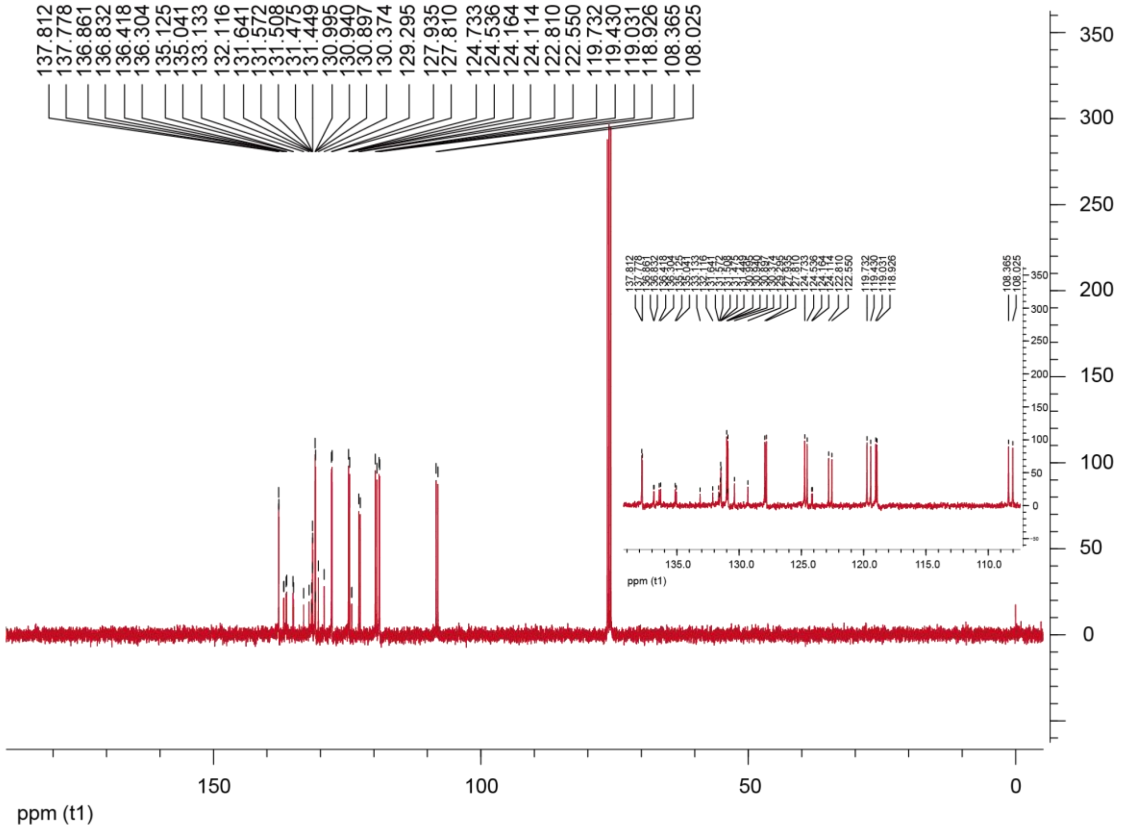

**Supplementary Figure 29**. $^{13}$C NMR (101 MHz) spectrum of DCzSBrSPO in $CDCl_3$.

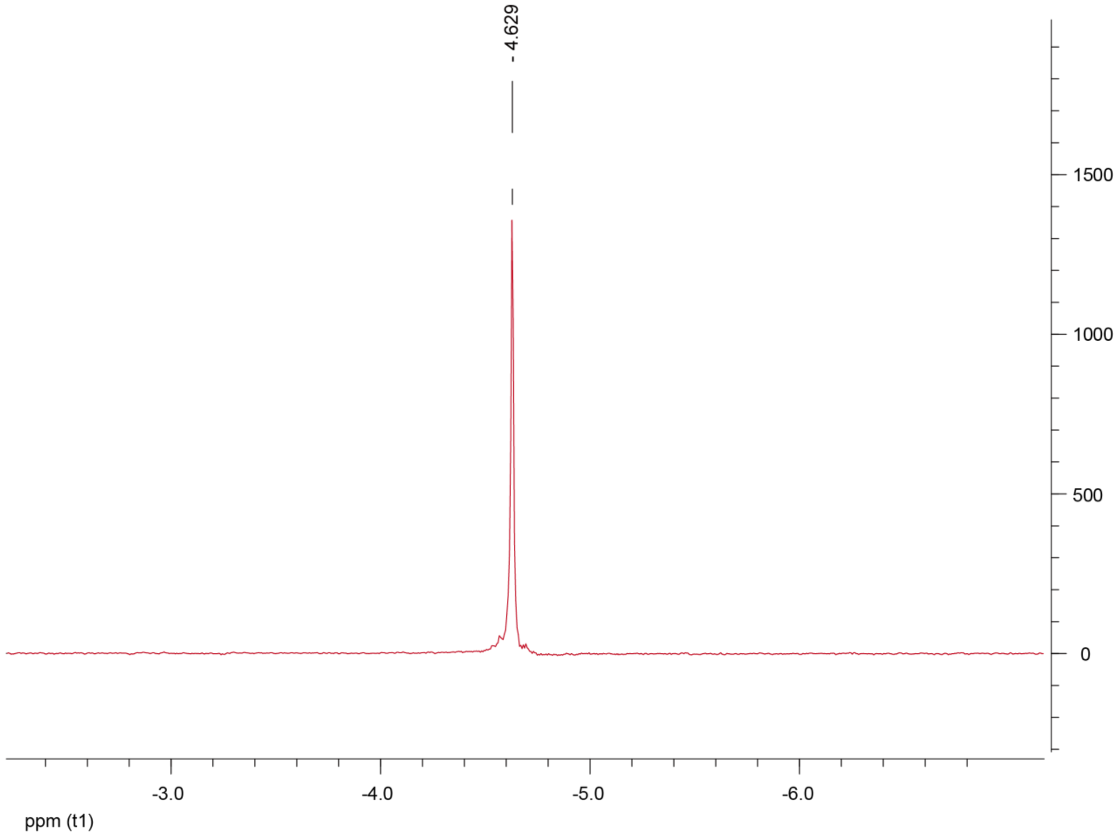


**Supplementary Figure 30**. $^{31}$P NMR spectrum of DCzSBrSP in $CDCl_3$.

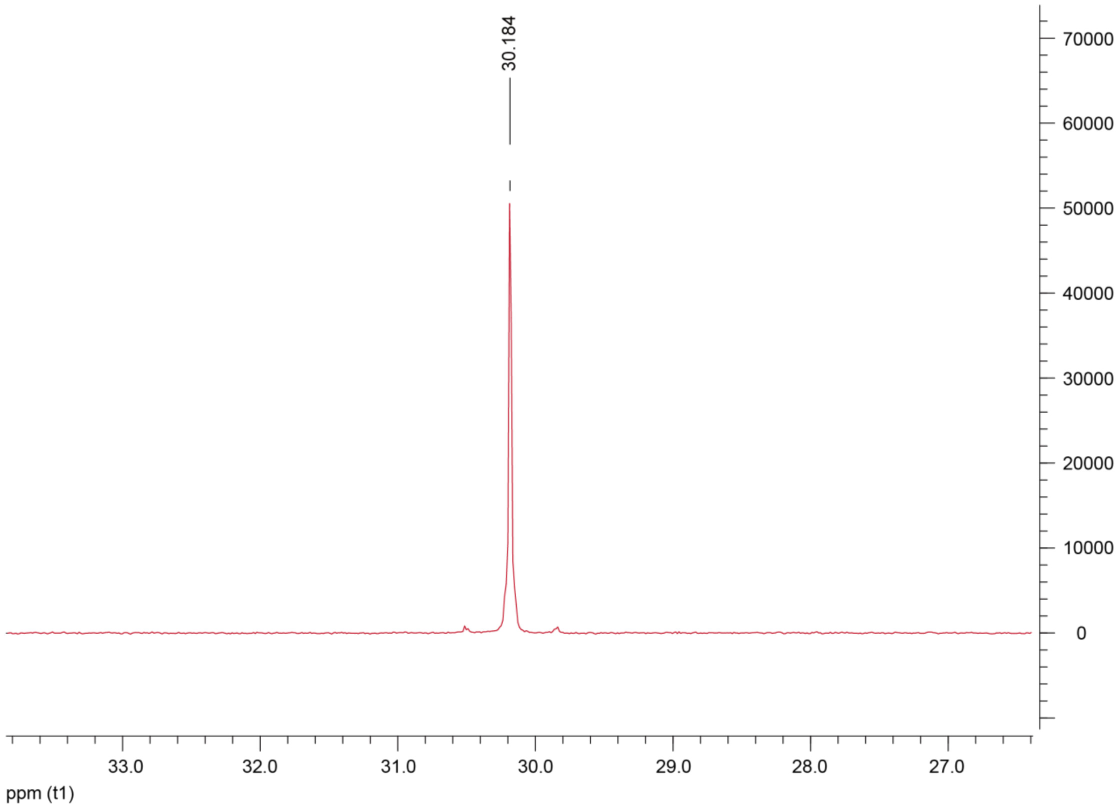


**Supplementary Figure 31**. $^{31}$P NMR spectrum of DCzSBrSPO in $CDCl_3$.

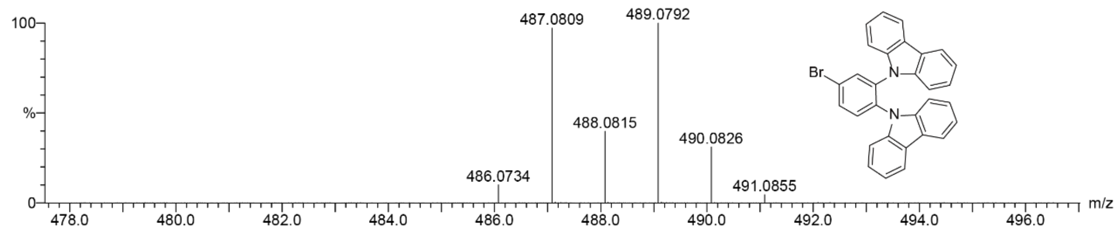


| | | | | | | | | |
|---|---|---|---|---|---|---|---|---|
| Minimum: | | | | -1.5 | | | | |
| Maximum: | | 5.0 | 10.0 | 50.0 | | | | |

| Mass | Calc. Mass | mDa | PPM | DBE | i-FIT | Norm | Conf(%) | Formula |
|---|---|---|---|---|---|---|---|---|
| 487.0809 | 487.0810 | -0.1 | -0.2 | 21.5 | 628.2 | n/a | n/a | C30 H20 N2 Br |

**Supplementary Figure 32**. HRMS spectra of DCzSBr.

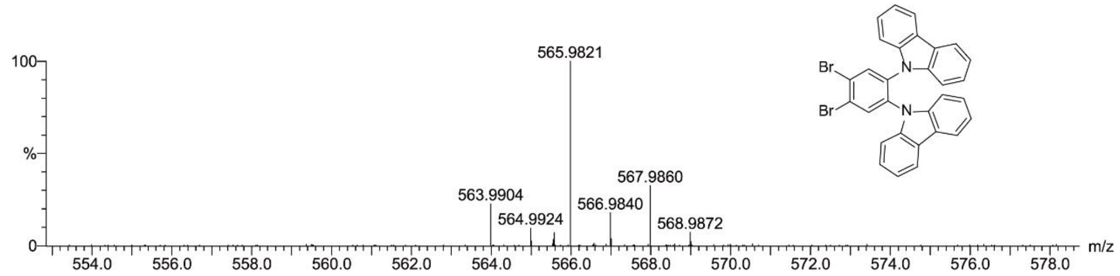


| | | | | | | | | |
|---|---|---|---|---|---|---|---|---|
| Minimum: | | | | -1.5 | | | | |
| Maximum: | | 5.0 | 20.0 | 50.0 | | | | |

| Mass | Calc. Mass | mDa | PPM | DBE | i-FIT | Norm | Conf(%) | Formula |
|---|---|---|---|---|---|---|---|---|
| 564.9924 | 564.9915 | 0.9 | 1.6 | 21.5 | 165.3 | n/a | n/a | C30 H19 N2 Br2 |

**Supplementary Figure 33**. HRMS spectra of DCzDBr.

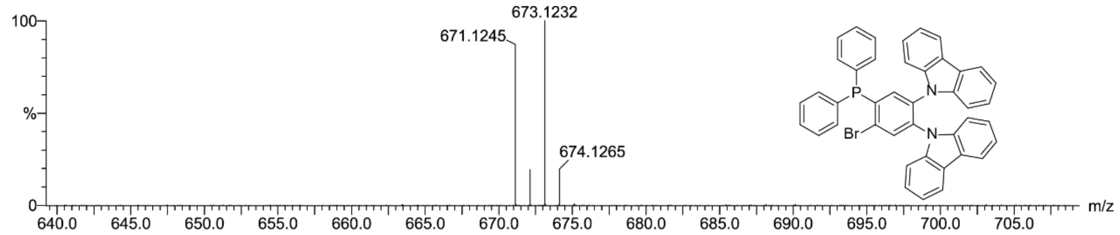


| | | | | | | | | |
|---|---|---|---|---|---|---|---|---|
| Minimum: | | | | -1.5 | | | | |
| Maximum: | | 5.0 | 20.0 | 50.0 | | | | |

| Mass | Calc. Mass | mDa | PPM | DBE | i-FIT | Norm | Conf(%) | Formula |
|---|---|---|---|---|---|---|---|---|
| 671.1245 | 671.1252 | -0.7 | -1.0 | 29.5 | 138.6 | n/a | n/a | C42 H29 N2 Br P |

**Supplementary Figure 34**. HRMS spectra of DCzSBrSP.

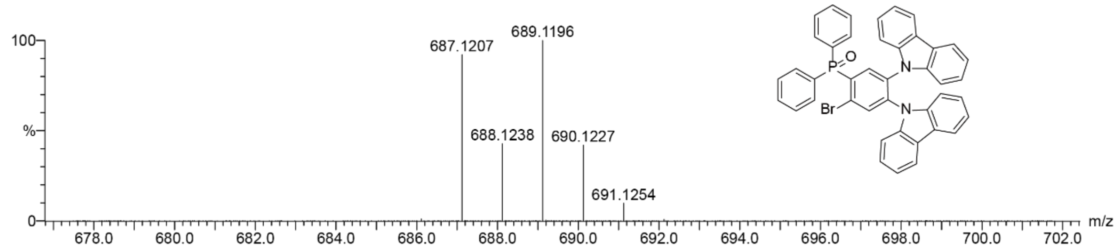


| | | | | | | | | |
|---|---|---|---|---|---|---|---|---|
| Minimum: | | | | -1.5 | | | | |
| Maximum: | | 5.0 | 10.0 | 50.0 | | | | |

| Mass | Calc. Mass | mDa | PPM | DBE | i-FIT | Norm | Conf(%) | Formula |
|---|---|---|---|---|---|---|---|---|
| 687.1207 | 687.1201 | 0.6 | 0.9 | 29.5 | 493.6 | n/a | n/a | C42 H29 N2 O Br P |

**Supplementary Figure 35**. HRMS spectra of DCzSBrSPO.

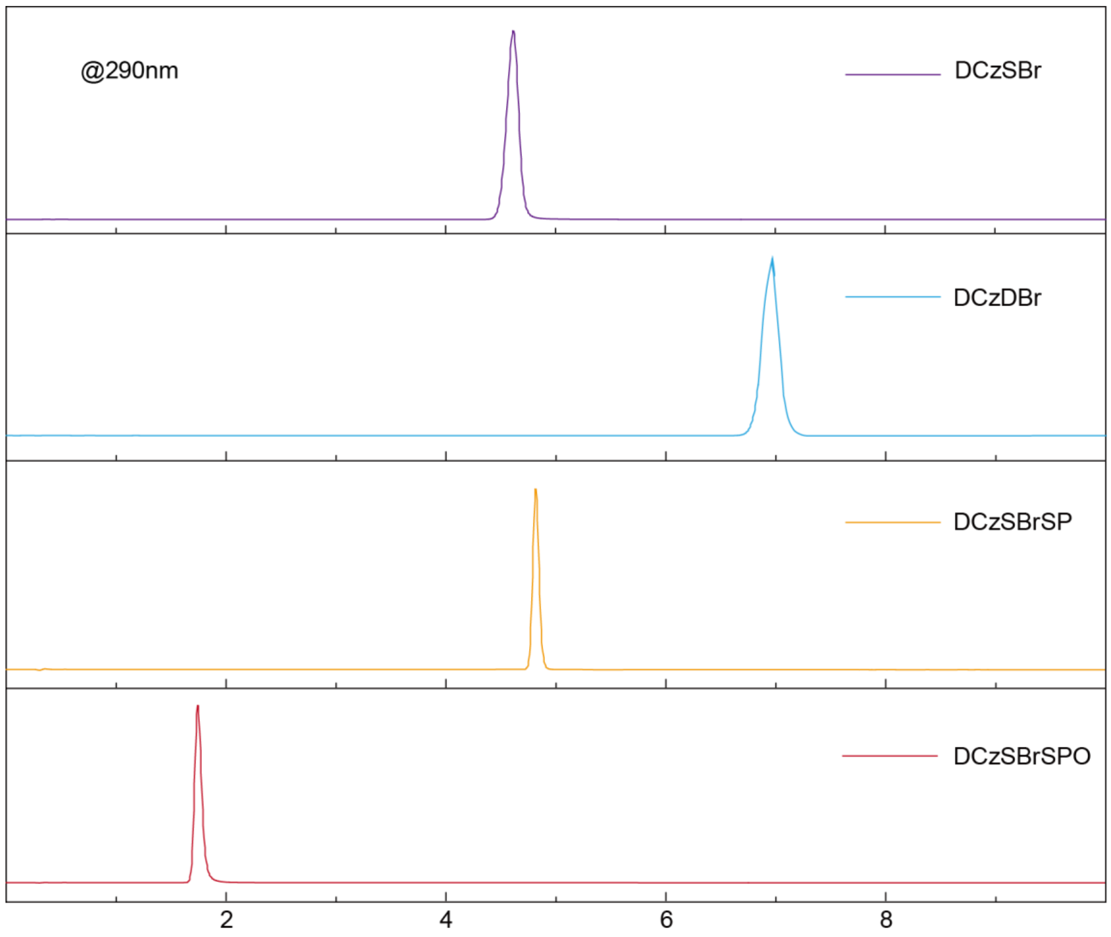


**Supplementary Figure 36**. HPLC spectra of DCzSBr, DCzDBr, DCzSBrSP and DCzSBrSPO. Mobile phases were acetonitrile/0.1% formic acid (75:25) for DCzSBr, DCzDBr and DCzSBrSPO, and acetonitrile/0.1% formic acid from 75:25 (0-5min) to 95:5 (5-10min) gradient elution for DCzSBrSP. UV detection at 290nm.

## Supplementary Note 7. References


1. Becke AD. Density-functional thermochemistry. III. The role of exact exchange. *J. Chem. Phys*. **98**, 5648-5652 (1993).
2. Lee C, Yang W, Parr RG. Development of the Colle-Salvetti correlation-energy formula into a functional of the electron density. *Phys. Rev. B* **37**, 785-789 (1988).
3. Martin RL. Natural transition orbitals. *J. Chem. Phys.* **118**, 4775-4777 (2003).
4. Frisch MJ, *et al.* Gaussian 09. D. 1 edn. Gaussian, Inc., Wallingford CT, USA (2009).
