# Supplementary material for: Delayed room temperature phosphorescence enabled by phosphines
Source: Nat Commun. 2024 May 2;15:3705. doi: 10.1038/s41467-024-47888-z (PMC11066103; doi:10.1038/s41467-024-47888-z)
Supplement: Supplementary file 3 — Description of Additional Supplementary Files [file 41467_2024_47888_MOESM3_ESM.pdf]

Description of Additional Supplementary Files:

Supplementary Movie 1: Variation of “HLJU logo” pattern prepared with IRTP and DRTP materials after UV excitation removal in 0.2 speeds, corresponding to Fig. 4a.

Supplementary Movie 2: Number changing procedure of “password panel” prepared with IRTP and DRTP materials after UV excitation removal in 0.2 speeds, corresponding to Fig. 4b.
